# Human three-dimensional in vitro model of hepatic zonation to predict zonal hepatotoxicity

**DOI:** 10.1186/s13036-019-0148-5

**Published:** 2019-03-06

**Authors:** Jaehwan Ahn, Jun-Ho Ahn, Seokjoo Yoon, Yoon Sung Nam, Mi-Young Son, Jung-Hwa Oh

**Affiliations:** 1 Department of Material Science and Engineering, Korea Advanced Institute of Science and Technology (KAIST), Daejeon, 34141 Republic of Korea; 2 Department of Predictive Toxicology, Korea Institute of Toxicology (KIT), Daejeon, 34114 Republic of Korea; 3 Stem Cell Research Center, Korea Research Institute of Bioscience and Biotechnology (KRIBB), Daejeon, 34141 Republic of Korea; 4 Department of Functional Genomics, KRIBB School of Bioscience, Korea University of Science and Technology (UST), Daejeon, 34113 Republic of Korea

**Keywords:** Liver zonation, Zonal hepatotoxicity, Alternative hepatic model, Drug screening, Wnt/β-catenin, CYP activities

## Abstract

**Background:**

Various hepatic models mimicking liver lobules have been investigated to evaluate the potential hepatotoxic effects of chemicals and drugs, but in vitro hepatic models of zonal hepatotoxicity have not yet been established. Herein, we developed a three-dimensional (3D) hepatic zonal channel to evaluate zone-specific hepatotoxicity. Based on the perivenous zone-3-like cytochrome P450 (CYP) expression patterns in metabolically active HepaRG cells treated with CHIR99021 (CHIR), which is an inducer of Wnt/β-catenin signaling, this culture model represents a novel tool for exploring hepatic zonation.

**Results:**

We generated and validated a 3D hepatic zonal channel model in which 3D HepaRG cells were well distributed in agarose hydrogel channels, and a linear gradient of CHIR was generated according to the zonal distance. According to the results from imaging analyses and bioanalytical experiments, acetaminophen (APAP) caused cytotoxicity in the zone-3 region of the 3D hepatic zonal channel, and the levels of nonphosphorylated β-catenin, CYP2E, and apoptotic proteins were remarkably increased in the zone-3-like region. Finally, the applicability of the 3D hepatic zonal channel model for the high-throughput screening of zonal hepatotoxicity was successfully evaluated using hepatotoxic drugs, including tamoxifen, bromobenzene, and APAP.

**Conclusions:**

The results indicated that tamoxifen induced cytotoxic effects, regardless of the zonal distance, while the zone-3-specific hepatotoxic drugs bromobenzene and APAP induced greater cytotoxic effects on cells in the zone-3-like region. This finding highlights the potential of our 3D hepatic zonation model as a valuable tool for replicating and evaluating zonal hepatotoxicity by mimicking the spatial features of liver lobules.

**Electronic supplementary material:**

The online version of this article (10.1186/s13036-019-0148-5) contains supplementary material, which is available to authorized users.

## Background

The liver plays key roles in homeostasis, including drug metabolism, and hepatic functions are highly specialized according to the spatial location along the portal-central vein axis of the liver lobule [1, 2]. The area from the portal triads to the hepatic vein is divided into three regions: zone-1, periportal area; zone-2, midzonal area; and zone-3, perivenous area. Hepatocytes exhibit functional heterogeneity in the liver lobule depending on their zonal location, which is known to be affected by the cellular microenvironment, such as blood oxygen, nutrient, and hormone concentrations [3, 4]. Hepatocytes located in the oxygen-rich zone-1 region perform hepatic functions, such as gluconeogenesis, β-oxidation, cholesterol synthesis and ureagenesis and are vulnerable to reactive oxygen species; however, these hepatocytes rapidly recover [5, 6]. Hepatocytes located in the zone-3 region express high levels of cytochrome P450, which metabolizes xenobiotic chemicals and performs functions such as lipogenesis, glycolysis, bile acid synthesis and glutamine synthesis [7, 8]. The compartmentalization of gene expression in the liver might underlie the ability of the tissue to control the pattern of zonal hepatotoxicity observed with some xenobiotics, such as acetaminophen (APAP), and several environmental agents [9].

Several studies have developed a reliable hepatic model to screen drug-induced hepatotoxicity. Monolayered hepatocytes, such as HepaRG and primary human hepatocytes, exhibiting drug-metabolizing activities have been used to screen hepatotoxicity, and three-dimensional (3D) hepatic spheroid models have recently been developed because these models exhibit more hepatic functions than a monolayer culture [10–12]. However, these hepatic models employ a homogenous population of hepatocytes, and therefore, an evaluation of zonal hepatotoxicity is difficult, which is indeed observed in vivo. Several studies aiming to develop an in vitro model of liver zonation that predicts zonal hepatotoxicity have been reported [13, 14]. Allen et al. designed a bioreactor system to generate an oxygen gradient and confirmed that cytochrome P450 (CYP) 2B levels were modulated by the oxygen gradient [14]. McCarty et al. developed a microfluidics system to mimic different hepatic functions according to the spatial location by modulating the gradients of exogenous hormones, including insulin and glucagon, and chemical induction agents. However, in vitro models that recapitulate the observed variations in the liver microenvironment and affect cell signaling and metabolic liver zonation are limited.

Oxygen and the β-catenin pathway are potential factors that modulate zonal metabolism in the liver [15, 16]. Oxygen is an electron acceptor in energy metabolism, and dissolved free oxygen concentrations are approximately 60–65 mmHg in the periportal blood and decrease to approximately 30–35 mmHg in the perivenous blood; this difference regulates zonal metabolism [4]. The dissolved free oxygen concentrations differ between periportal and perivenous blood vessels in vivo, and this difference in concentrations according to spatial zonation are rarely controlled in vitro. However, an oxygen gradient alone is not sufficient to reflect zonal metabolism in hepatocytes [17, 18]. Wnt/β-catenin signaling is known to be a key signaling pathway that regulates the expression of drug-metabolizing enzymes [19, 20]. Wnt/*β*-catenin signaling was also highlighted as a key endogenous regulator of the expression of drug-metabolizing enzymes, such as hepatic CYPs [21]. CHIR99021 (CHIR), a highly selective inhibitor of glycogen synthase kinase-3 (GSK3β), is a strong inducer that activates Wnt/β-catenin signaling [22]. In this study, we attempted to induce the differential expression and activation of drug-metabolizing enzymes to mimic zonal liver metabolism by modulating Wnt/β-catenin signaling. A gradient of CHIR concentrations generated zonal toxicity responses in HepaRG cells by inducing the expression and activity of zone-3-specific CYPs.

To the best of our knowledge, this study is the first to establish an in vitro model of hepatic zonation by modulating the zonal expression of genes encoding Phase I drug-metabolizing enzymes in hepatocytes by regulating Wnt/β-catenin signaling. Based on these findings, we successfully designed and validated a novel 3D hepatic zonal channel model in which the zonal hepatotoxicity of compounds is detected using direct imaging analyses and bioanalytical experiments. By evaluating drug-induced zonal hepatotoxicity, this 3D biomimetic hepatic zonation system represents a novel tool for elucidating liver zonation and its physiological and pathophysiological implications.

## Results

### Modulation of the activities of drug-metabolizing cytochrome P450 enzymes by a CHIR treatment

**Fig. 1:**
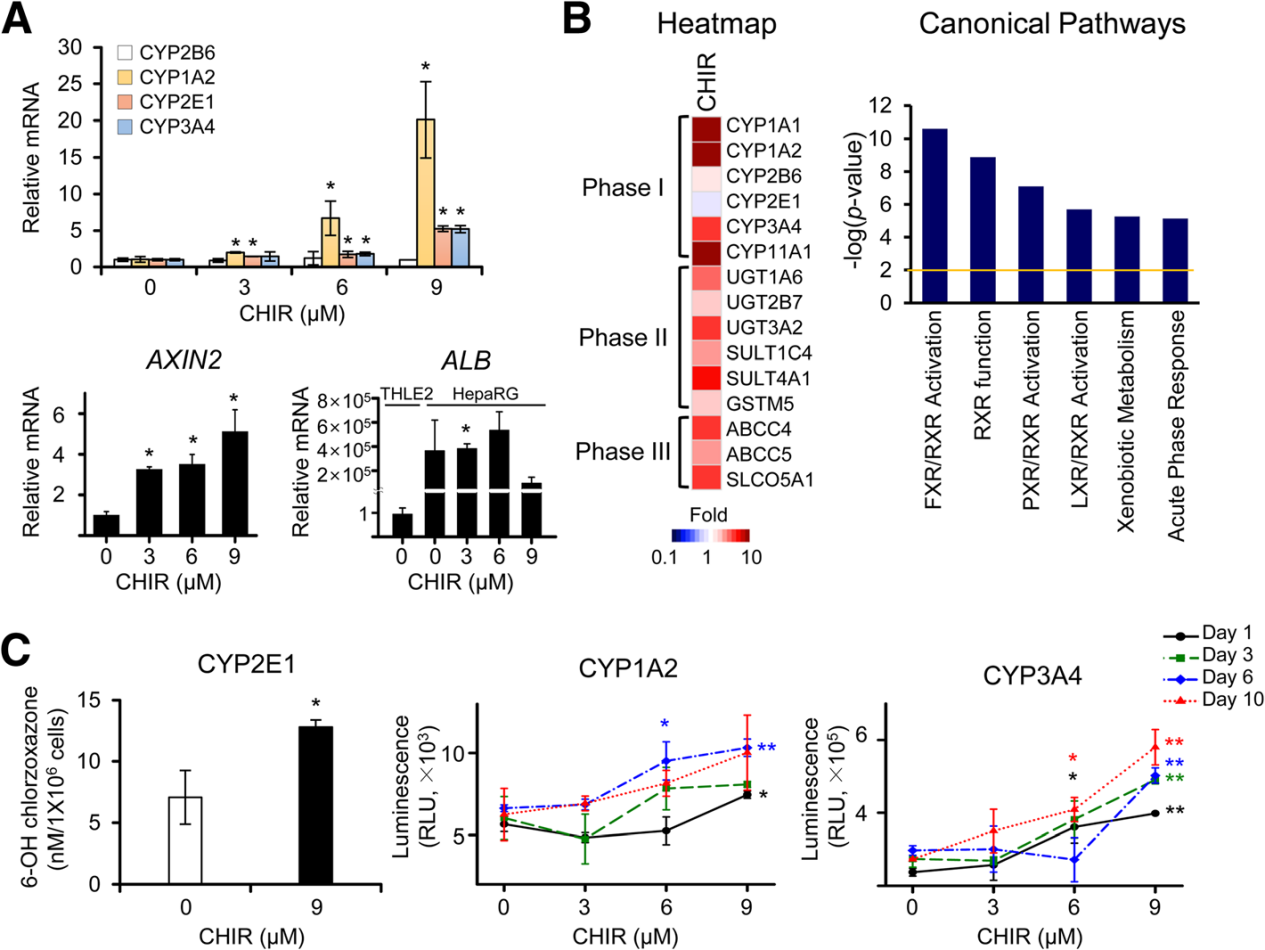
Changes in the expression and activity of CYP enzymes in HepaRG cells induced by the CHIR treatment. The expression of the CYP mRNAs and enzymatic activities of CYPs (CYP2B6, CYP1A2, CYP2E1, and CYP3A4) were analyzed in CHIR-treated HepaRG cells. **a** Fully differentiated HepaRG cells were exposed to various concentrations of CHIR. The relative levels of *CYPs*, *AXIN2*, and *ALB* (albumin) mRNAs in HepaRG cells were examined after 3 days of CHIR treatment using qRT-PCR. The relative level of *ALB* was calculated in the HepaRG cells before and after CHIR treatment comparing with THLE2 cells. The basal expression level of *ALB* mRNA in HepaRG cells was remarkably greater than that of THLE2 cells (**b**) A microarray analysis was performed using HepaRG cells that had been treated with 9 μM CHIR for 3 days. The heatmap of genes involved in drug metabolism was analyzed using Gene-E software, and canonical pathways of differentially expressed genes (2-fold, *P* < 0.01) were analyzed using IPA software. **c** Enzymatic activities of CYPs in HepaRG cells treated with CHIR for up to 10 days. The activities of CYP1A2 and CYP3A4 were measured using the P450-Glo CYP assay, and the CYP activity values were normalized to cell number by dividing the P450-Glo luminescence values by the CCK-8 absorbance values, according to the manufacturer’s instructions. CYP2E1 activity was measured by determining the conversion of chlorzoxazone to OH-chlorzoxazone using HPLC-tandem mass spectrometry. All data are presented as the means ± SD. *P < 0.05 and **P < 0.01 compared with the untreated group at each time point; Student’s *t*-test

We focused on zonal characteristics of CYP heterogeneity and first evaluated the viability of HepaRG cells and the mRNA expression levels of human CYP isoforms after treatment with different concentrations of CHIR. The fully differentiated HepaRG cells were treated with CHIR for 10 days, and the medium supplemented with an appropriate concentration of CHIR was replaced every 3 days. In the cell viability assay using CCK-8 reagents, the relative viability of HepaRG cells was slightly increased in a dose-dependent manner during the 10 days of treatment with CHIR (Additional file 1: Figure S1A) and hepatocyte-like morphology was observed in HepaRG cells before and after treatment with CHIR (Additional file 1: Figure S1B). This CHIR-induced increase in the viability of HepaRG cells is consistent with the findings of a previous investigation showing that CHIR induced the proliferation of human cardiomyocytes by upregulating β-catenin expression [23]. After exposure to various concentrations of CHIR for 3 days, the mRNA levels of several isotypes of CYPs, including *CYP2B6*, *CYP1A2*, *CYP2E1*, and *CYP3A4,* were measured using qRT-PCR. *CYP2B6* is generally expressed in the liver without spatial specification [24], and *CYP1A2*, *CYP2E1*, and *CYP3A4* are predominantly expressed in the zone-3 region of the hepatic lobule [25]. Interestingly, as the CHIR concentration increased, the levels of the *CYP2E1* and *CYP3A4* mRNAs increased 5-fold compared with the untreated control cultures of differentiated HepaRG cells (Fig. 1a). Remarkably, *CYP1A2* mRNA expression was strongly induced, reaching a 20-fold maximal effect after treatment with 9 μM CHIR. The expression of *CYP2B6* was not noticeably changed by any treatment condition. Based on these findings, at concentrations greater than 6 μM, CHIR induces the transcription of specific CYP subfamily members, which are expressed in perivenous hepatocytes in zone-3, in a dose-dependent manner. In other human hepatocytes, including normal THLE2 and cancerous Huh7 cell lines, significant changes in *CYP2E1* and *CYP3A4* expression were not observed in THLE2 cells, and a 3 day CHIR treatment only increased the level of the *CYP2E1* mRNA in Huh7 cells (Additional file 2: Figure S2). We found that CHIR more efficiently induced CYP expression in metabolically competent HepaRG cells than in normal THLE2 hepatocytes and Huh7 hepatocarcinoma cells. Levels of the *AXIN2* mRNA, a representative target gene of β-catenin, were increased in response to treatment with CHIR in a dose-dependent manner, showing that CHIR activated Wnt/β-catenin signaling in HepaRG cells (Fig. 1a). We also confirmed that the levels of *ALB* (albumin) mRNA, a marker of hepatic function, were remarkably increased in CHIR-treated HepaRG cells than in THLE2 controls (Fig. 1a).

To evaluate the molecular changes in HepaRG cells in response to treatment with CHIR, we analyzed the global changes in gene expression using a microarray experiment. DEGs (differentially expressed genes) were identified by filtering to select genes with an at least 2-fold change in expression that was statistically significant (*P* < 0.01) compared to the control, resulting in the identification of 3058 DEGs in cells treated with CHIR. As shown in the heatmap of the genes related to drug metabolism (Phase I, II and III), most of the Phase I CYPs, including *CYP1A1*, *CYP1A2*, and *CYP3A4*, and several Phase II enzymes and Phase III transporters were overexpressed in the CHIR-treated group (Fig. 1b). Unlike the qRT-PCR results, *CYP2E1* expression was slightly decreased in the microarray, which may be due to the use of a different probe region (for exon 7) than the primer region (for exon 11) used in the qRT-PCR. The canonical pathways of DEGs were analyzed using IPA. Genes related to xenobiotic metabolism, including FXR/RXR, RXR, PXR/RXR, and LXR/RXR functions, were selected as key pathways that were differentially regulated in the CHIR-treated group (Fig. 1b).

Additionally, we assessed the activities of CYP2E1, CYP1A2, and CYP3A4, which are specific CYPs expressed in zone-3, in HepaRG cells treated with serial concentrations of CHIR for up to 10 days (Fig. 1c). The enzymatic activities of perivenous region-specific CYP1A2, CYP2E1, and CYP3A4 were remarkably increased in HepaRG cells treated with CHIR. Their expression levels peaked in cells treated with 9 μM CHIR. Collectively, the CHIR treatment increased the activities of several CYP isotypes, which is similar to the phenomenon observed in the perivenous region (zone-3).

### Generation of the zonal drug toxicity responses of HepaRG cells treated with CHIR

**Fig. 2:**
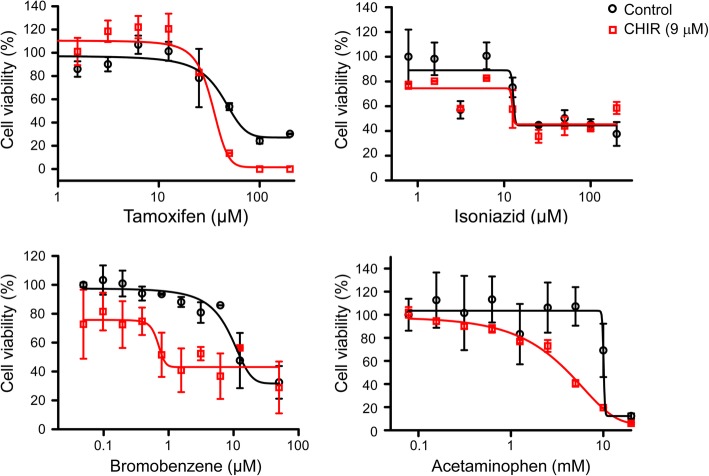
Cell viability of HepaRG cells treated with four hepatotoxic drugs after CHIR pretreatment. The hepatotoxic drugs tamoxifen, isoniazid, bromobenzene, and APAP were administered to HepaRG cells pretreated with CHIR for 3 days. After 3 days of hepatotoxic drug treatment, the cell viability of HepaRG cells was measured using the CCK-8 assay, and the dose-response curve was analysed using GraphPad Prism software

We next evaluated the cytotoxic effects of hepatotoxic drugs in HepaRG cells after pretreatment with or without CHIR. Differentiated HepaRG cells were pretreated with or without 9 μM CHIR and the viability was examined using a CCK-8 assay on day 2 after treatment with four different hepatotoxic drugs. Tamoxifen, bromobenzene, isoniazid, and APAP were used as hepatotoxic drugs, and these drugs form toxic intermediates through the actions of Phase I enzymes. Tamoxifen and isoniazid are CYP3A4-mediated hepatotoxic drugs, whereas bromobenzene and APAP are CYP2E1- and CYP1A2-mediated hepatotoxic drugs. In the histopathological observations of a rat model derived from the publicly available Open TG-GATEs database, tamoxifen and isoniazid induce hepatotoxic effects across the overall region of the hepatic lobule, while bromobenzene and APAP cause hepatotoxicity in the perivenous region of zone-3 (Additional file 3: Figure S3). The viability of four hepatotoxic drugs was evaluated in HepaRG cells at 3 days after pretreatment with 9 μM CHIR to mimic the microenvironment of zone-3. CHIR-treated HepaRG cells exhibited different patterns of dose-response curves for each drug; these patterns could be used to classify drugs into two groups, sensitive or insensitive to the CHIR concentration. The cytotoxic effects of tamoxifen and isoniazid were similar for untreated control and CHIR-treated HepaRG cells (Fig. 2, upper panels). However, the cytotoxic effects of bromobenzene and APAP increased in cells treated with 9 μM CHIR (Fig. 2, lower panels). The IC_50_ values of the four hepatotoxic drugs indicated that the cytotoxic effects of bromobenzene and APAP were significantly different, depending on CHIR concentration (Table 1). The results demonstrated that the CHIR could induce the zonal toxicity responses in HepaRG cells by modulating the expression and activity of CYP2E1 and CYP1A2.

**Table 1:** The cytotoxic concentration of hepatotoxic drugs in CHIR-treated HepaRG cells

Hepatotoxic drugs	Untreated control		9 μM CHIR	
	IC_50_	95% confidence interval	IC_50_	95% confidence interval
Tamoxifen (μM)	51.0	34.3–67.7	35.2	25.5–44.8
Isoniazid (μM)	13.3	12.5–14.1	13.2	12.1–14.3
Bromobenzene (μM)	12.5	9.3–15.7	0.8^*^	0.4–1.2
Acetaminophen (mM)	10.0	9.7–10.3	4.2^*^	3.2–5.2

*P*-values indicate significant differences between the IC_50_ values of hepatotoxic drugs after CHIR treatment. * *P* < 0.05

### Development of a 3D biomimetic hepatic zonation system

**Fig. 3:**
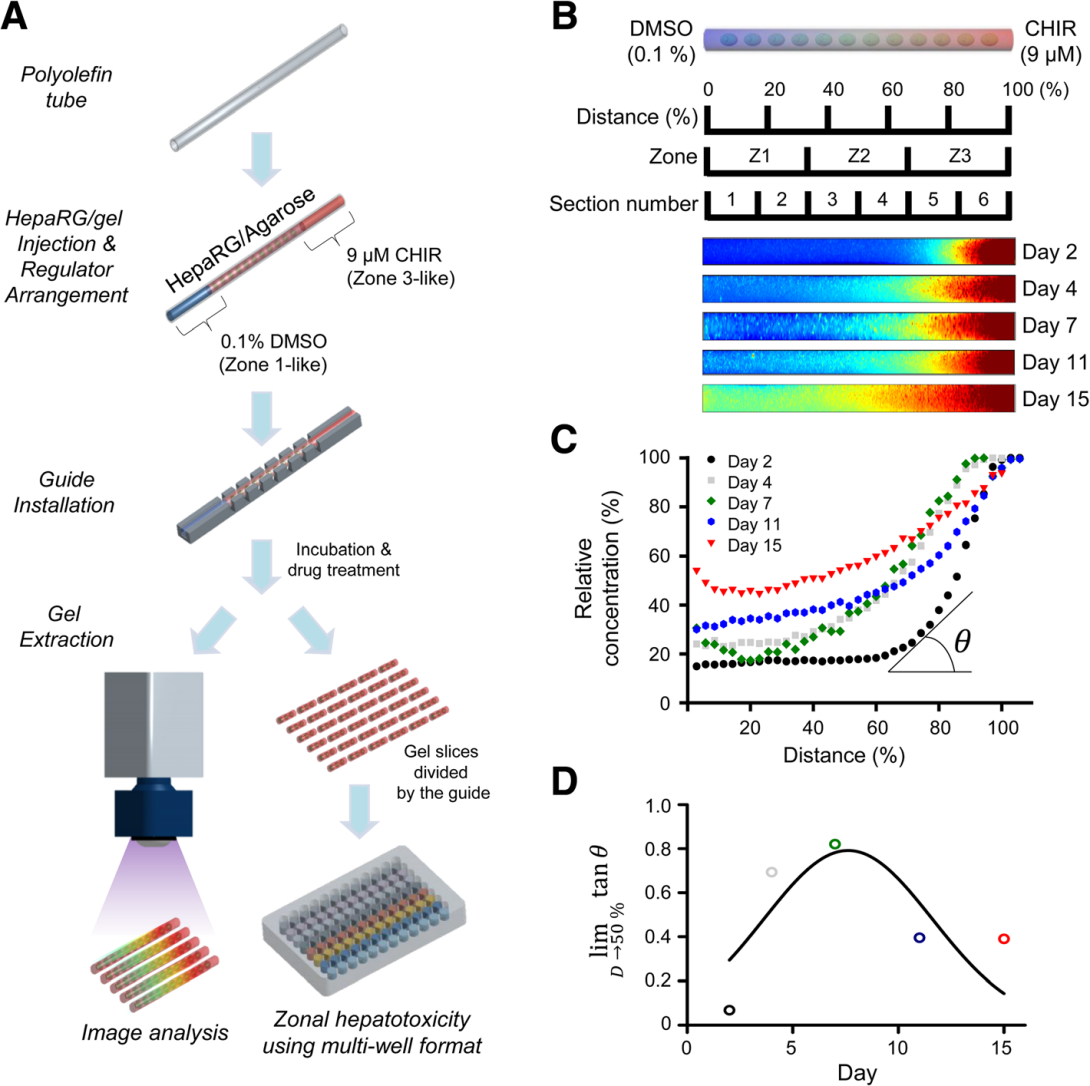
Development of the in vitro 3D hepatic zonation platform. **a** Schematic flow of the zonal hepatotoxicity evaluation system using the 3D hepatic zonal channel model. An agarose hydrogel gel containing HepaRG cells was injected into the polyolefin tube, and 3D HepaRG cells were cultured in the gel matrix for 7 days. For natural diffusion, 0.1% DMSO and 9 μM CHIR were located at the left (Inlet 1) and right side (Inlet 2) of the channel, respectively. The 3D hepatic zonal channel can be sliced into several pieces representing zone-1-like, zone-2-like and zone-3-like sections with the installed guide and then treated with hepatotoxic chemicals. **b** The relative CHIR diffusion in the 3D hepatic zonal channel was monitored for up to 15 days by UV illumination using the ChemiDoc XRS+ Imaging System. The intensity of CHIR across the 3D hepatic zonal channel was imaged using the MATLAB program. **c** The relative intensity of CHIR according to the distance from Inlet 1 to Inlet 2 was quantified using the MATLAB program and plotted. **d** The intensity-concentration curves for each time point were analysed with the curve-fitting method, and the linearity was calculated from the limit (lim) of tangent Ɵ approaching 50% of the distances. A tangent value close to 1 indicates the most linearity. On day 7, the CHIR concentration profile was close to a linear diffusion gradient in the 3D hepatic zonal channel

We designed a 3D hepatic zonal channel that mimics the microenvironment of liver zonation and is capable of screening zonal hepatotoxicity. A schematic of the procedure is presented in Fig. 3a. Briefly, an agarose hydrogel containing differentiated HepaRG cells was injected into the biocompatible polyolefin tube and HepaRG cells were encapsulated in an agarose hydrogel and then grown in a 3D structure in a polyolefin tube for 7 days. CHIR was linearly diffused in the polyolefin tube containing 3D cultures of HepaRG cells for 7 days and the test chemicals were then added to the intact or sliced pieces of the 3D hepatic zonal channel. Finally, zonal toxicity was evaluated in vitro using an imaging analysis. We first evaluated the cell viability and distribution of 3D HepaRG cultures in the polyolefin tube (Additional file 4: Figure S4). During the long-term culture for 11 days, the cell viability and distribution of 3D HepaRG cultures were examined on days 3, 6, and 11 using confocal imaging after staining with dual live/dead cell dyes. The confocal microscopy images of the top, cross, and side sections showed that the 3D cultures of HepaRG cells in the agarose hydrogel were well distributed in the polyolefin tubes (Additional file 4: Figure S4A). The viability of HepaRG cells was not significantly affected by the 3D culture conditions in the agarose hydrogel compared with the conventional monolayer 2D culture conditions (Additional file 4: Figure S4B). The analysis of live and dead cells showed that a high proportion of live cells was maintained on day 6, but the proportion of dead cells increased on day 11 (Additional file 4: Figure S4C). In the case of long-term cultivation using the 3D model, the necrotic cores caused by limited oxygen diffusion is a critical issue [26]. According to a previous study, 3D HepaRG spheroids less than 200 μm in size were suitable for long-term culture for up to 21 days without forming necrotic cores [11]. However, high-throughput screening using 3D hepatic spheroid and scaffold model have been performed at less than 7 days because the variations in size, viability and functionality are difficult to control [27, 28]. The results from the present study showing a high proportion of viable cells up to day 6 are comparable to long-term cultivation in the previous investigations.

To generate a 3D hepatic zonal channel, we introduced CHIR at one end (Inlet 2) of the tube and culture medium at the opposite end (Inlet 1) of the polyolefin tube (Fig. 3a). CHIR diffused through agarose hydrogel channels containing 3D HepaRG from Inlet 2 (9 μM CHIR with medium containing 0.1% DMSO) towards Inlet 1 (medium containing 0.1% DMSO). The 3D hepatic zonal channel was separated by the installed guide to divide the channel into a zone-1-like section (the pericentral region) and a zone-3-like section (the perivenous region). We optimized the time of diffusion, which can provide the linear gradient of CHIR in the 3D hepatic zonal channel from Inlet 2 to Inlet 1 (Fig. 3b-d). The diffusion behavior of CHIR through agarose hydrogel channels was visualized using a UV illuminator, and the CHIR concentration in images was analyzed using MATLAB software (Fig. 3b). CHIR possesses an aromatic ring that absorbs UV light and emits at 305 nm. In a previous study, the diffusion coefficient of rhodamine B (479 g mol^− 1^), which has a similar molecular weight to CHIR (465.34 g mol^− 1^), was calculated in a 1% agarose hydrogel [29], and the relative diffusion coefficient was estimated based on the ratio of the molecular weights.

$$ \frac{D_{CHIR}}{D_{RhB}}=\sqrt[3]{\frac{Mw_{RhB}}{Mw_{CHIR}}} $$

We assumed that diffusion through the agarose hydrogel channel occurs in one dimension, and the diffusion solute has a spherical geometry with a radius proportional to the molecular weight. The diffusion coefficient of rhodamine B is approximately 2.96 × 10^− 6^ cm^2^/s, and the similar diffusion coefficient of CHIR in the 3D hepatic zonal channel was estimated to be 2.98 × 10^− 6^ cm^2^/s. The CHIR concentration at various times of day was quantified using MATLAB and plotted (Fig. 3c). The intensity-concentration curves were analyzed with a curve-fitting method and the linearity was calculated based on the tangent at 50% of the distance according to the period to determine the optimum diffusion time for CHIR to develop a linear gradient across the zonal channel (Fig. 3d). The results demonstrated that 7 days is the optimum period to obtain a linear gradient of CHIR in the 3D hepatic zonation model.

### Application of the 3D hepatic zonation model to evaluate zonal hepatotoxicity in vitro

**Fig. 4:**
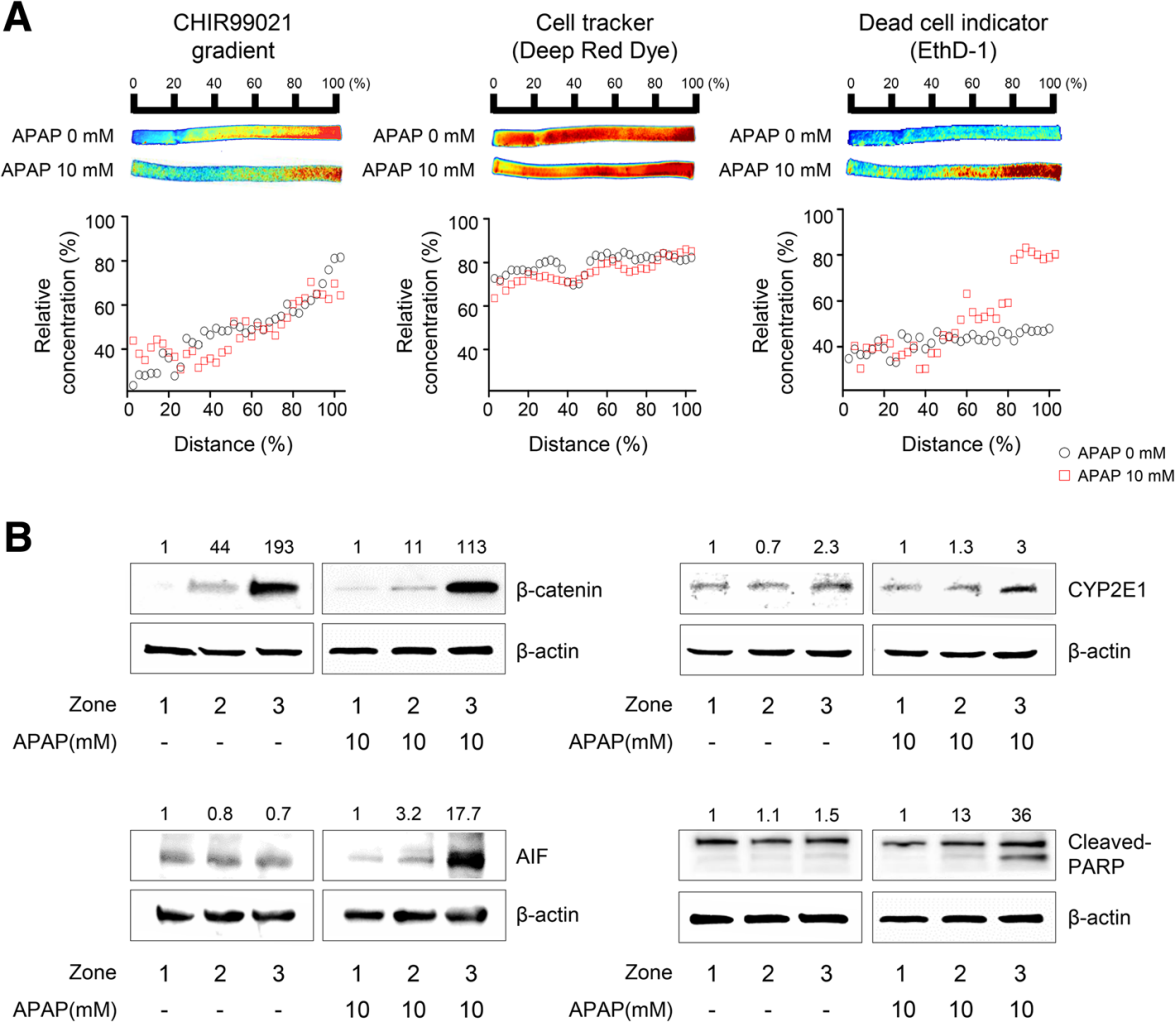
Evaluation of the in vitro zonal hepatotoxicity using the 3D hepatic zonation model. **a** Application of the 3D hepatic zonation model using imaging analysis after APAP treatment. The intact 3D HepaRG sample in the agarose hydrogel channel was removed from the polyolefin tube and then treated with 10 mM APAP for 2 days in a multi-well plate. Quantitative analysis of 3D hepatic zonal channels was performed using staining with dual live/dead dyes as well as CHIR for diffusion of CHIR, CellTracker Deep Red Dye for cellular distribution, and EthD-1 for membrane-damaged cytotoxic cells, and the ChemiDoc XRS+ Imaging System, and images were visualized and analysed with MATLAB. The red and blue colours indicate high and low intensity of the dyes, respectively. The relative concentration was quantified and plotted for the control and APAP-treated groups. The EthD-1^+^-damaged cells were predominantly observed in the high CHIR concentration region in response to 10 mM APAP treatment. **b** Molecular experimental approaches using the 3D hepatic zonation model. The 3D HepaRG sample was removed from the 3D hepatic zonal channel and then divided into 3 pieces corresponding to zone-1-like, zone-2-like and zone-3-like regions according to the guide. After treatment with 10 mM APAP for 2 days, total protein was extracted from the 3D HepaRG and western blot analysis was performed. The expression of β-catenin, CYP2E1, AIF, and cleaved PARP was analysed, and β-actin was used as an internal control

To demonstrate the practical utility of our 3D hepatic zonal channel model, we first applied it to evaluate zonal hepatotoxicity. HepaRG cells in polyolefin tubes were treated with test chemicals in the well-based culture platform. After treatment with the test chemicals, a 3D hepatic zonal strip comprising HepaRG cells in polyolefin tubes was sliced into several pieces and labeled as distance (%), zone or section number, depending on the user’s evaluation methods, such as live imaging, cell viability assays, or biosample analyses. APAP, a zone-3-specific hepatotoxic drug, was used to quantify zonal hepatotoxicity in the 3D hepatic zonal channel model using an imaging analysis (Fig. 4a). After 7 days of CHIR diffusion in the zonal channel, the intact 3D hepatic zonal channel extracted from the tube was treated with APAP for 2 days, directly imaged, and evaluated using a ChemiDoc system after staining with dual live/dead cell dyes. We confirmed the distribution of CHIR in the 3D hepatic zonal channel to compare the proportion of live and dead cells according to the gradient of CHIR, and the distribution profile of CHIR after 7 days of diffusion in the zonal channel showed that CHIR diffused in a manner that led to a concentration gradient (Fig. 4a, left panels). HepaRG cells were prestained with CellTracker Deep Red fluorescent probes before being loaded in the polyolefin tubes. HepaRG cells were uniformly dispersed throughout the agarose hydrogel channel in the polyolefin tubes, regardless of the distance (Fig. 4a, middle panels). After a 10 mM APAP treatment for 2 days, the cytotoxic effects on 3D HepaRG cells were evaluated with EthD-1 staining, an indicator of cellular membrane damage. In the region with a high CHIR concentration mimicking zone-3, 3D HepaRG cells were more severely damaged by the APAP treatment than at the point in the channel farther from the initial diffusion point of CHIR (Inlet 2) (Fig. 4a, right panels). These data indicated that zonal hepatotoxicity can be quantitatively evaluated using a 3D hepatic zonal channel model by imaging analysis.

The 3D hepatic zonal channel permits molecular experimental approaches, such as western blotting, using the 3D HepaRG cells recovered from the channel. The 3D HepaRG sample was recovered from the 3D hepatic zonal channel and then divided into 3 pieces corresponding to the zonal regions, according to a distance guide. After treatment with 10 mM APAP, we purified the proteins from 3D HepaRG, and the expression levels of several target proteins involved in APAP metabolism and hepatotoxicity were evaluated by western blotting (Fig. 4b). The non-phosphorylated β-catenin expression was higher in the zone-3-like region than in the zone-1- or zone-2-like regions, indicating that GSK3β was effectively inhibited by CHIR in both the untreated and APAP-treated channels (Fig. 4b, top left panels). The level of CYP2E1 increased 2- to 3-fold in the zone-3-like region compared to the zone-1- or zone-2-like regions (Fig. 4b, top right panels). The Wnt/β-catenin pathway regulates CYP2E1 transcription, and the results showed that CYP2E1 expression was coordinately regulated by the increase of active β-catenin. APAP induces apoptotic cell death in hepatocytes through a signaling cascade characterized by an increase in mitochondrial dysfunction, AIF (apoptosis-inducing factor) release and PARP (poly (ADP-ribose) polymerase) cleavage [30]. Using the 3D hepatic zonal channel, we confirmed that APAP induced apoptotic cell death by increasing AIF expression and PARP cleavage in the zone-3-like region, where nonphosphorylated β-catenin and CYP2E1 levels were increased (Fig. 4b, lower panels). Thus, the 3D hepatic zonal channel model was efficiently used for imaging and molecular experiments to evaluate zonal hepatotoxicity.

**Fig. 5:**
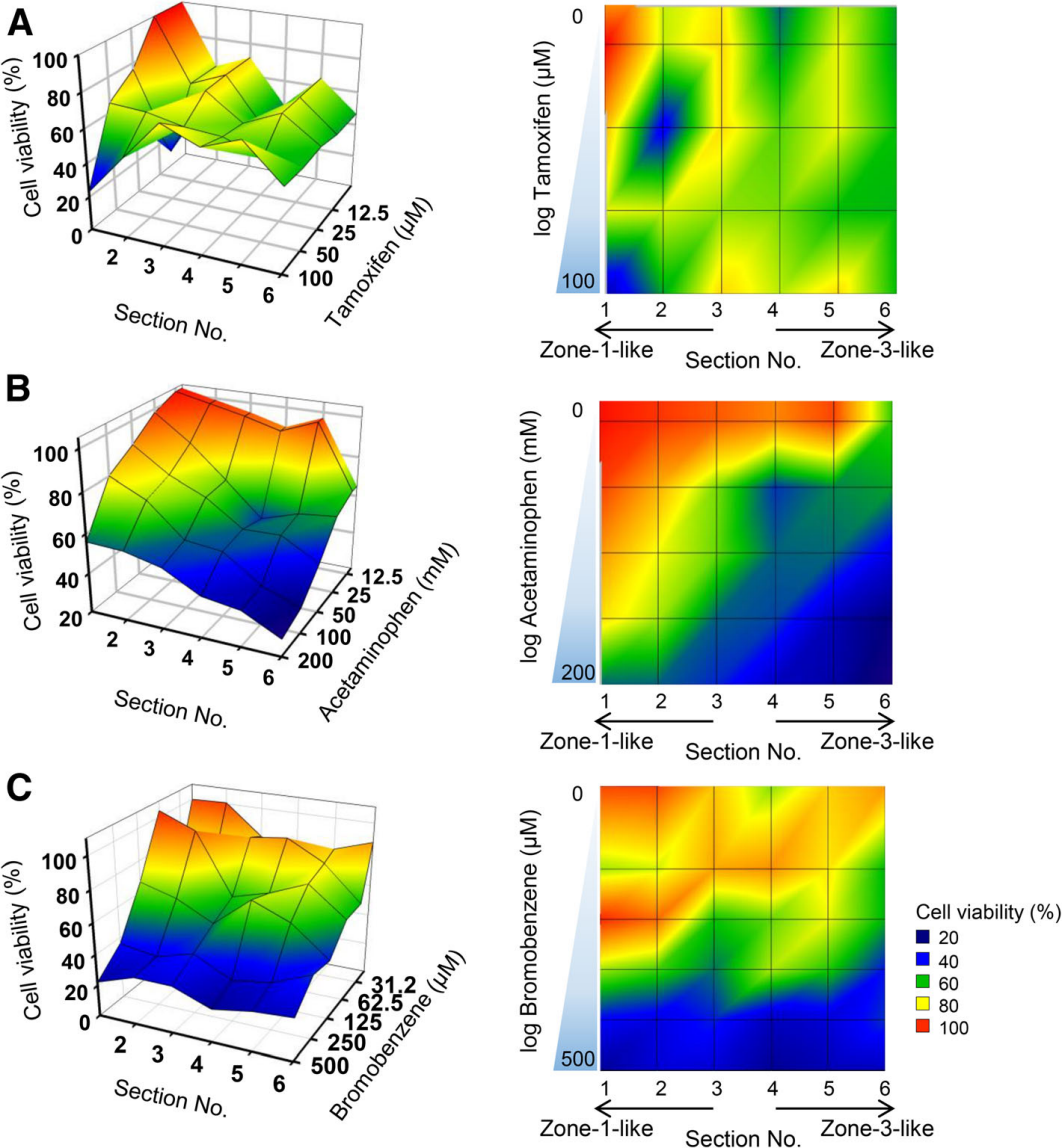
Imaging profiles of zonal hepatotoxicity using the 3D hepatic zonal channel model. Three different hepatotoxic drugs, tamoxifen (**a**), APAP (**b**), and bromobenzene (**c**), were administered to the sectioned 3D HepaRG sample with an CHIR gradient for 2 days, and cell viability was then evaluated as described in the Materials and Methods section. The values of the x-, y- and z-axes indicate the section number (from 1 to 6), cell viability, and drug concentration, respectively. The imaging profiles display the relative cell viability according to the colour legend. The red and blue colours indicate higher and lower cell viability, respectively

Furthermore, we examined the applicability of the 3D hepatic zonal channel model in screening assays, preferably high-throughput screening of zonal hepatotoxicity. Three different hepatotoxic drugs, tamoxifen, bromobenzene, and APAP, were applied to this system. The 3D hepatic zonal channel with a CHIR gradient was divided into six sections according to the distance guide and was treated with various concentrations of hepatotoxic drugs for 3 days. The viability of 3D HepaRG cells in each zonal section was evaluated using the CCK-8 assay. The relative cell viability was analysed according to the concentrations of each hepatotoxic drug and zonal sections using the MATLAB program (Fig. 5). These results showed that tamoxifen caused toxicity in 3D HepaRG cells in all sections, regardless of zonal specificity (Fig. 5a). However, the APAP treatment led to a remarkably different pattern of cell viability according to the distance and APAP concentration. The 3D HepaRG cells tended to be more vulnerable to the effects of APAP when they were located closer to the zone-3-like section (high CHIR concentration region) (Fig. 5b). In addition, the profile of cell viability after bromobenzene treatment was similar to that of APAP (Fig. 5c). However, 3D HepaRG cells in the hepatic zonal channel model were more susceptible to treatment with APAP than bromobenzene. Under 100 μM bromobenzene, 3D HepaRG cells experienced greater cytotoxic effects in zone-3-like sections than in zone-1-like sections, but there were similar cytotoxic effects at high bromobenzene concentrations over 100 μM. These results indicated that hepatotoxic drugs causing zonal hepatotoxicity can be efficiently screened and can be classified as zone-specific hepatotoxic drugs by our 3D hepatic zonal channel model.

## Discussion

Identifying potential hepatotoxic chemicals is a challenging task. Hepatotoxicity is one of the most critical findings that necessitates further investigations of new drugs in clinical trials [31]. Although animal studies are mandatory in nonclinical studies, a retrospective analysis revealed that animals do not precisely predict hepatotoxicity in humans, with only approximately 45–55% of predictions identified as correct [32]. Hepatotoxicity is mostly linked to drug metabolism in the liver, and the low concordance of hepatotoxicity can be caused by the difference in drug metabolism or hepatic physiology between animals and humans [33, 34]. Although human primary hepatocytes and HepaRG cells exhibiting drug-metabolizing functions have been mostly used in nonclinical studies [10, 35], hepatic zonation showing different drug-metabolizing activities is another hurdle in predicting hepatotoxicity. Hepatic functional markers, such as albumin and transferrin, are widely expressed across the hepatic lobule without zonal specificity, while zonal dependence occurs for drug metabolism as well as glutamine and glucose metabolism [36]. The heterogeneous activities of drug-metabolizing enzymes depending on the zonal location can cause different adverse effects on hepatic lobules, and the evaluation of zonal hepatotoxicity is a considerable challenge. In nonclinical studies, human primary hepatocytes are considered the gold standard model for evaluating drug metabolism, but these cells exhibit severe limitations in terms of interdonor variability, functional instability and propagation of long-term cultures [37]. In the present study, we used metabolically competent HepaRG cells, which have been used as a complement to primary hepatocytes to generate the hepatic zonal system and retain hepatic functions, such as CYP enzymatic activity, albumin/transferrin production, and glutamine/glucose metabolism [38]. We generated a gradient of CYP enzymes in functional HepaRG cells by modulating Wnt/β-catenin signaling to mimic the zonation dependence of drug metabolism, and this system classified hepatotoxic drugs that exhibit zonal toxicity.

To date, a few trials have generated liver zonation in vitro using a bioreactor or a microfluidics system by regulating oxygen, exogenous hormones or chemicals, but these factors could not implement zonation for drug metabolism [13, 14]. The hepatic artery is located near the portal vein, and when the blood flows from the portal vein (zone-1) to the central vein (zone-3), an oxygen gradient can be generated across the hepatic lobule, and oxygen-enriched blood can induce oxidative stress in zone-1 [18]. Although oxidative stress conditions slightly decreased CYP activity, the change was not sufficient to generate a gradient of CYP enzymes. We have also evaluated the CYP activity in HepaRG cells by adding H_2_O_2_, an inducer of oxidative stress, at concentrations up to 200 μM, which are not cytotoxic to HepaRG cells (Additional file 5: Figure S5A). The fully differentiated HepaRG cells were treated with H_2_O_2_ and the enzymatic activities of CYP1A2 and CYP3A4 were significantly decreased in HepaRG cells following exposure to H_2_O_2_ (Additional file 5: Figure S5B). However, no significant differences in the cytotoxic effects of the four hepatotoxic drugs were observed on HepaRG cells subjected to pretreatment with or without H_2_O_2_ (Additional file 5: Figure S5C). Thus, H_2_O_2_ might mimic the oxidative stress conditions in the pericentral region (zone-1) showing the low activity of CYPs, but H_2_O_2_ does not appear to play key roles in mimicking the zonal toxicity in vitro regarding drug metabolism.

Apart from oxygen, McCarty et al. reported microfluidics systems that generate a zonal function of glycogen uptake and urea formation with an insulin/glucagon mixture, but there were limitations in maintaining hepatic function with drug metabolism capacity over time in the microfluidics platforms [13]. Although human primary hepatocytes are comparably active in drug metabolism, their activities rapidly decrease in monolayer cultures over time, and the activities of drug-metabolizing enzymes are difficult to maintain in vitro [39]. Since 3D cultures permit hepatocytes to maintain more functionality during long-term cultivation than monolayer cultures, we cultured metabolically competent HepaRG cells in a 3D structure by encapsulating them in agarose hydrogels, which maintain the cellular phenotypes with higher cell viability and drug metabolism activity than monolayer cultures [40]. In the case of HepaRG cells, higher expression of functional markers, such as albumin, has been observed in 3D cultures, including spheroid and scaffold models, compared with 2D cultures [11, 28]. In the 3D hepatic zonal channel reported in the present study, the agarose hydrogel had a high mechanical strength, allowing the channel to maintain a stable shape and to be divided into small pieces for analyses of biosamples, implying that our 3D hepatic zonal system may be suitable for screening subacute responses to hepatotoxic drugs that occur over several days in vivo with hepatic zonation.

As shown in the present study, the 3D hepatic zonal channel screened the zonal hepatotoxic drugs APAP and bromobenzene and the nonzonal hepatotoxic drug tamoxifen. In particular, this system identified the zonal hepatotoxic drugs that were metabolized by CYP1A2, CYP2E1, and CYP3A4, which are zone-3 specific CYPs. Meanwhile, in Fig. 2, the cytotoxic effects of tamoxifen and isoniazid, CYP3A4-mediated hepatotoxic drugs, were not different between the HepaRG cells treated with and without CHIR, although we confirmed that CHIR increased the CYP3A4 activity. CYP3A4 is the main drug-metabolizing enzyme for tamoxifen but CYP2D6 and CYP2C19 are also involved in metabolizing this drug into the reactive 4-hydroxy metabolite [41]. Isoniazid is also metabolized by CYP2C9/2C19, CYP2D6, CYP2E1, and CYP3A4 [42]. Based on these findings, the 3D hepatic zonal channel might be limited to screening zonal hepatotoxicity in vitro for drugs that are metabolized through the complex interactions of drug metabolizing enzymes.

The 3D hepatic zonal system strongly supports the concept of generating hepatic zonation by modulating Wnt/β-catenin signaling. Several hypotheses about the mechanisms underlying liver zonation have been suggested, and Wnt/β-catenin signaling is an important driver of drug metabolism [7]. Mouse liver displayed a distribution of β-catenin in the perivenous region, and the localization of β-catenin is tightly regulated by canonical Wnt pathways [43]. Following the loss of expression of CYP enzymes, particularly Cyp2e1 and Cyp1a2, in *Ctnnb1* knockout mice [44], Wnt/β-catenin signaling controls the expression of CYP enzymes. Despite the findings concerning the association of Wnt/β-catenin signaling with drug metabolism, the in vitro zonal model generated by modulating Wnt signaling had not been developed because the drug-metabolizing activities of hepatocytes are vulnerable to culture conditions, and thus a gradient of CYP enzymes is difficult to generate in vitro*.* In the present study, the expression of *AXIN2*, a β-catenin target gene, was increased in a dose-dependent manner in CHIR-treated HepaRG cells with a functional Wnt/β-catenin signaling pathway. CHIR is generally used to activate the Wnt/β-catenin signaling pathway by inhibiting the GSK3β, which phosphorylates β-catenin and induces its proteosomal degradation [22]. However, Gerbal-Chaloin et al. reported that the expression of CYP2E1 and CYP1A2, but not CYP3A4, is regulated by Wnt/β-catenin signaling [25]. According to Briolotti et al., CHIR also activates the AhR (aryl hydrocarbon receptor) and PXR (pregnane X receptors) signaling pathways, as a partial agonist of AhR and PXR [45], as well as the Wnt/β-catenin pathway. In the 3D hepatic zonal channel, we postulated that CYP2E1 and CYP1A were activated by CHIR in a Wnt/β-catenin-dependent manner, but CYP3A4 might be activated in a Wnt/β-catenin-independent manner through cross-talk with AhR or PXR signaling. The more precise mechanisms by which CHIR alters drug metabolism in the 3D hepatic zonal channel require further investigation.

Human stem cell-derived hepatocytes, hepatic organoids or 3D spheroid models have been suggested as alternative hepatic models to mimic the hepatic physiology in humans and enable a more precise prediction of hepatotoxicity in vivo. However, these models exhibit homogenous drug metabolism activities without zonal specificity and are not efficient at screening zonal hepatotoxic compounds [46, 47]. The proposed 3D hepatic zonal systems enable the use of high-throughput approaches to screen and classify zonal hepatotoxic drugs through a direct imaging analysis. This system also provides molecular insights into the response of the recovered zone-specific hepatocytes to zonal hepatotoxicity. The zonal hepatotoxic profiles showing the response of compounds to drug-metabolizing enzymes are essential for developing an in silico model of hepatotoxicity. Furthermore, the zonal function of drug metabolism was generated by CHIR, and the defined condition can be used to generate additional hepatic zonal models by applying the system to human stem cell- and induced pluripotent stem cell-derived hepatocytes or hepatic organoid models. These approaches will further lead to risk assessments for personalized toxicology accounting for the heterogeneity of drug metabolism.

## Conclusions

In the present study, we generated a 3D hepatic zonal system by modulating Wnt/β-catenin signaling. Although a few in vitro models mimicking liver zonation have been reported, limitations regarding the implementation of hepatic zonation to assess drug metabolism remain. This system provides a simple and robust method to generate the zonal distribution of drug metabolism and permit screens of zonal hepatotoxic drugs and subacute toxicity over several days. The zonal toxicity profiles provide information that addresses the conflicting data on heterogeneous drug metabolism and clarifies the spatial heterogeneity of toxicological responses. This information should be helpful for developing predictive models of hepatotoxicity and for decision-making in drug development processes.

## Methods

### Cells and chemicals

Undifferentiated human HepaRG cells (HPR101) were purchased from Biopredic International (Rennes, France) and human normal immortalized THLE2 and hepatoma Huh7 cells were obtained from American Type Culture Collection. William’s E medium with no glutamine, bronchial epithelium growth medium (BEGM) medium with complete supplement, Dulbecco’s modified Eagle medium (DMEM), fetal bovine serum (FBS), fibronectin, phosphate buffered saline (PBS), L-glutamine, penicillin/streptomycin, and trypsin-EDTA were purchased from Thermo Fisher Scientific (Rockford, IL, USA). Collagen type I was purchased from Advanced Biomatrix (San Diego, CA, USA). Insulin, hydrocortisone hemisuccinate, dimethyl sulfoxide (DMSO), methanol, acetonitrile anhydrous, CHIR, 30% wt H_2_O_2_ solution, and agarose (low gelling temperature) were purchased from Sigma-Aldrich (St. Louis, MO, USA). Chlorzoxazone, tamoxifen, isoniazid, bromobenzene, and APAP were acquired from Sigma-Aldrich (USA).

### Cell culture

The undifferentiated human HepaRG cells were cultured in Williams’ E medium containing 10% serum, 1% L-glutamine, 1% penicillin/streptomycin, 5 μg mL^− 1^ insulin, and 50 μM hydrocortisone. The cells were cultured on 150 cm^2^ T-flask culture plates at 37 °C, 5% CO_2_, and 100% humidity for 2 weeks, and the medium was changed every 2–3 days. After 2 weeks, the culture medium was replaced with culture medium containing 2% DMSO to activate differentiation. The culture medium with 2% DMSO was renewed every 2–3 days. After 2 weeks, the differentiated cells were washed with PBS two times and rinsed with trypsin-EDTA for 1 min. Then, the cells were obtained with trypsin-EDTA. THLE2 cells were cultured in complete BEGM medium on the collagen/fibronectin coated plate and Huh7 cells were maintained in DMEM, supplemented with 10% heat-inactivated FBS and 1% penicillin/streptomycin.

### Cell viability assay and CYP activity measurement

Differentiated cells were seeded in a 96-well clear bottom plate at a density of 100,000 viable cells per well. HepaRG cells were cultured for another 7 days to ensure the complete differentiation of the monolayer, according the manufacturer’s instructions. After 7 days, the differentiation medium was replaced with culture medium containing 0.1% DMSO to reduce the basal CYP levels. After 3 days, each group of HepaRG cells was exposed to various concentrations of CHIR (control, 3 μM, 6 μM, or 9 μM), and the medium with the concentrated chemicals was replaced every 3 days. At certain times (day 1, 3, 6, or 10), Cell Counting Kit-9 (CCK-8) assays (Dojindo, Japan) and P450-Glo CYP assays (Promega, WI, USA) were performed according to the manufacturer’s instructions after two washes with PBS. The CYP activity results were normalized to the cell number by dividing the P450-Glo luminescence values by the CCK-8 absorbance values. CYP2E1 enzyme activity was evaluated after CHIR treatment. Because there is no commercially available CYP2E1 measurement kit, CYP2E1 activity was evaluated by measuring the transformation of chlorzoxazone to the 6-hydroxychlorzoxazone metabolite. The differentiated HepaRG cells were cultured on 6-well plates in growth medium containing 0.1% DMSO to decrease the basal CYP level. The CYP2E1 activity assay was performed on day 3. After medium removal, 500 μL of 300 μM chlorzoxazone in growth medium containing 0.1% DMSO was added and kept in the incubator for 6 h. A total of 400 μL of the solution was collected and diluted (1:1) with acetonitrile with an internal standard for the conversion reaction. 6-Hydroxychlorzoxazone was assessed by HPLC tandem mass spectrometry. The system consisted of an AB Sciex Qtrap 4000 (AB Sciex, Foster City, CA, USA) mass spectrometer interfaced with an Agilent 1200 series HPLC system (Agilent Technologies, Palo Alto, CA, USA). Chromatography was performed at 35 °C with a 5 μL injection volume using a Knetex C18 column (50 mm × 2.1 mm, 5 μm particle size; Phenomenex, Torrance CA, USA) at a flow rate of 0.3 mL min^− 1^. The two mobile phases were 0.1% formic acid in ultrapure water and acetonitrile.

### Quantitative RT-PCR (qRT-PCR) and microarray analysis

Fully differentiated HepaRG cells cultured on the 12-well collagen-coated culture plate in the 0.1% DMSO culture medium at least 3 days, THLE2, and Huh7 cells were treated with CHIR (control, 3 μM, 6 μM, 9 μM) for 3 days. Following incubation, RNA samples were extracted by a Maxwell 16 system for RT-PCR. We used BLAST to design primer sequences and purchased the sequences from Macrogen (Seoul, Korea). The sequences of the primers are presented in Additional file 6: Table S1, and 18S rRNA was used as an internal control. One-step reverse transcription was performed using a Power SYBR Green RNA-to CT 1-Step kit according to the manufacturer’s instructions.

For the microarray analysis, HepaRG cells treated with 0.1% DMSO and 9 μM CHIR for 3 days were purified using an RNeasy mini kit (Qiagen, Valencia, CA, USA). An Agilent Human GE 4 K microarray was used for this experiment (Agilent Technologies, USA). The synthesis of target cRNA probes and the hybridization were performed using Agilent’s Low RNA Input Linear Amplification Kit (Agilent Technologies, USA) according to the manufacturer’s instructions and as described previously [48, 49]. Briefly, 1 μg of total RNA was used and amplified, and labelled cRNA was purified on the cRNA Cleanup Module (Agilent Technologies, USA). The labelled cRNA target was quantified using an ND-1000 spectrophotometer (NanoDrop Technologies, Wilmington, DE, USA). After checking the labelling efficiency, fragmented cRNA was hybridized into Agilent’s arrays at 65 °C for 17 h using an Agilent Hybridization oven (Agilent Technologies, USA). The hybridized arrays were washed according to the manufacturer’s washing protocol (Agilent Technologies, USA). Preprocessing and data normalization were performed using Agilent’s GeneSpring GX. DEGs with at least 2-fold changes and statistically significant differences in expression (*P* < 0.01) compared with that of the controls were selected. Gene function was analysed using Ingenuity Pathways Analysis (IPA) software (Qiagen, USA). The statistical significance of canonical pathways was calculated by a right-tailed Fisher’s exact test.

### Cell viability assay

All stock solutions of the drug were prepared in DMSO. The differentiated HepaRG cells were prepared in 96-well plates containing growth medium with 0.1% DMSO for at least 3 days. The cells were treated with growth media containing 0.1% DMSO or 9 μM CHIR. After 3 days, the cells were washed with PBS and immediately exposed to two-fold diluted concentrations of tamoxifen (200 μM), bromobenzene (50 μM), isoniazid (200 μM), or APAP (20 mM) for 2 days on culture days. After washing with PBS, cell viability was assessed using a CCK-8 assay according to the manufacturer’s protocol. The absorbance was measured using a Promega GloMax luminometer (Mannheim, Germany). The dose-response curves were analysed based on nonlinear regression by four-parameter equations, and the 50% inhibitory concentration (IC_50_) was calculated using GraphPad Prism software (GraphPad, San Diego, CA, USA).

### Fabrication of the 3D hepatic zonal channel

Twenty milligrams of agarose, low melting temperature, was dissolved in 1 mL of Williams’ E medium containing 2% L-glutamine, 2% penicillin/streptomycin, 10 μg mL^− 1^ insulin, and 100 μM hydrocortisone at 90 °C for 2 h. Additionally, the differentiated cells that had been cultured in growth medium with a low concentration of DMSO for 3 days were obtained with trypsin-EDTA. A total of 500 μL of a 100,000 cells per mL suspension was mixed with an agarose solution, which was slightly cooled before use, in the ratio of one to one (final concentration of agarose gel: 1%). A total of 360 μL of the cell suspension was quickly injected into the 5 mm diameter and 9 cm long polyolefin tube with a 3D printed guide, which can help to divide the sample into 3 or 6 parts after cultivation. The tube and its guide were placed in an incubator for 1 h for gelation. To generate diffusion of CHIR, growth medium containing 0.1% DMSO was injected into the left side of the tube (Inlet 1), 9 μM CHIR in growth medium containing 0.1% DMSO was injected into the right side of the tube (Inlet 2) by a 1 mL syringe, and the media were renewed every day. As shown in Fig. 3b, the diffusion of CHIR was tracked for up to 15 days by the ChemiDoc XRS+ Imaging System (Bio-Rad, Hercules, CA, USA) after the gel was extracted from the tube. The relative concentrations of CHIR and the linear diffusion were calculated based on the saturated maximum intensity. The intensity along the cross-section of the hydrogel after tube removal was obtained using MATLAB software (MathWorks, Natick, MA, USA). On day 7, when the diffusion profiles showed linearity, the hydrogel containing cells and mimicking a zone-specific microenvironment was extracted from the tube for image analysis and sliced into 3 or 6 parts along the guide for western blotting or drug screening.

### Confocal imaging analysis

The 3D HepaRG cells were extracted from the 3D hepatic zonal channel on day 3, 6, and 11. Cell viability of 3D HepaRG cells was also evaluated by calcein-acetoxymethyl (Calcein-AM) and ethidium homodimer-1 (Ethd-1) dual-staining assays. The dye solution was aspirated, and the cells were incubated for 1 h. The green fluorescence (excitation/emission wavelengths: 494/517 nm) and the red fluorescence (excitation/emission wavelengths: 528/617 nm) were monitored using a confocal microscope (LSM 800 with Airyscan; Zeiss, Jena, Germany) as described previously [50]. To determine the 3D morphology of HepaRG cells in the hydrogel matrix, a series of z-stack confocal microscopy images covering a 190 μM depth of the hydrogel fractions was projected in perspective view.

### Image analysis of zonal hepatotoxicity using the 3D hepatic zonal channel

To monitor the 3D distribution of HepaRG cells encapsulated in an agarose hydrogel, CellTracker™ Deep Red probes were loaded into the HepaRG cells according to the manufacturer’s instructions. After the dyeing step, the 3D hepatic zonal channel was prepared as described above. After 7 days of CHIR diffusion, the 3D hepatic zonal channel was removed from the tube by gentle air blowing with a 1 mL pipette without slicing and immediately soaked in 2 mL of serum-free media without APAP or with 10 mM APAP for 2 days in a 6-well plate. After two washes with PBS, the treated 3D hepatic zonal channels were stained with 2 μM EthD-1, an indicator of the membrane-damaged cells, to indicate cell death by the hepatotoxic drug. Image analysis was performed by the ChemiDoc system after two washes with PBS (CellTracker™ Deep Red; 695/55 filter/red epi-illumination, CHIR; standard filter/UV transillumination, EthD-1; 605/50 filter/green epi-illumination). The intensity along the cross-section of the 3D hepatic zonal channel after tube removal was obtained using MATLAB software as explained above.

### Protein extraction from the hepatic zonal channel and western blot analysis

3D hepatic zonal strips treated by APAP were sliced into 3 pieces as zone-1-, zone-2- and zone-3-like sections. The average weight of a piece of the channel was approximately 90 mg. We added 40 μL of 5× RIPA buffer, 45 μL of 4× Laemmli sample buffer and 5 μL of mercaptoethanol immediately. The sample was heated on a 90 °C block at 500 rpm for 10 min and then instantly moved to a 42 °C heat block. Two units of thermostable β-agarase (Nippon Gene, Japan) in buffered aqueous glycerol solution was added for 10 min to dissolve the agarose matrix and kept for another 10 min to degrade agarose in the strip. Cell debris and undegraded agarose were removed by centrifugation at 120,000 rpm for 10 min. Protein quantification was performed by measuring the intensity of β-actin-HRP antibody (sc-47,778, Santa Cruz, TX, USA) after the primary western blot analysis. Then, 30 μL of the supernatant was loaded with equal amounts of the solution (40 μL) in the wells in an acrylamide gel along with molecular weight markers, and gel electrophoresis was performed at 80 V for 2 h. The transfer process was then performed at 100 V for 1.5 h. The protein-transferred membranes were blocked in 5% skim milk in Tween Tris-buffered saline (TTBS) at room temperature for 1 h. Non-phoso-β-catenin, CYP2E1, apoptosis-inducing factor (AIF), and poly ADP ribose polymerase (PARP) were used to detect the protein profile in the secondary western blot with quantified samples and incubated overnight in the solution with primary antibody against the target protein after three washes with Tris-buffered saline (TBS). The information of the antibodies are presented in Additional file 7: Table S2. After incubation, the secondary antibodies were conjugated to the primary antibody for detection. Finally, the Clarity™ Western ECL Substrate was used for detection.

### In vitro zonal hepatotoxicity screening using the 3D hepatic zonal channel

After 7 days of CHIR diffusion, the tube and hydrogel were sliced into 6 sections at the same time, and the section closest to the CHIR source was labelled section No. 6, with the numbers for the other sections decreasing in order by location. The CHIR-gradient 3D HepaRG cells were treated with two-fold diluted concentrations of tamoxifen (0–100 μM), APAP (0–200 mM), and bromobenzene (0–500 μM) for 48 h at 37 °C and 5% CO_2_. After washing with PBS, cell viability was measured using a CCK-8 assay according to the manufacturer’s protocol. The absorbance at 450 nm was measured using a Promega GloMax luminometer (Mannheim, Germany). Cell viability was calculated as the percentage of viable cells relative to the untreated controls. The patterns of zonal hepatotoxicity are presented based on relative cell viability using the MATLAB software as described above.

### Statistical analysis

All data are presented as the means ± SD for cell viability, mRNA levels, and enzymatic activity of CYPs. Statistically significant differences between groups were analyzed using Student’s *t*-test with GraphPad Prism software (GraphPad, USA). *P* < 0.05 indicated a statistically significant difference between groups.

## Additional files

Additional file 1: **Figure S1.** Viability of monolayer-cultured HepaRG cells after CHIR treatment (days 1, 3, 6, and 10). (A) Cell viability was evaluated using CCK-8 assays on days 1, 3, 6, and 10 after the CHIR treatment. (B) HepaRG cells was observed before and after 3 days of CHIR treatment under a phase-contrast microscope (scale bar, 100 μm). The microscopic observation showed that fully differentiated HepaRG cells were organized in small clusters and displayed a typical hepatocytes-like morphology. (TIF 2598 kb)

Additional file 2: **Figure S2.** qRT-PCR of CYP enzymes in various hepatocyte cell lines after CHIR treatment. The hepatocytes, including THLE2 (A) and Huh7 (B) were treated with CHIR for 3 days. The expression levels of zone-3-specific CYPs, such as *CYP2E1* and *CYP3A4*, were evaluated by qRT-PCR. (TIF 532 kb)

Additional file 3: **Figure S3.** Histopathologic observation of liver from rats treated with hepatotoxic drugs. The light microscopic image of the tamoxifen-, isoniazid-, bromobenzene-, and APAP-treated liver was obtained from the Open TG-GATEs database. The Sprague-Dawley rats were orally administered each drug for 29 days; tamoxifen, 20 mg/kg; isoniazid, 200 mg/kg; bromobenzene, 100 mg/kg; and APAP, 1000 mg/kg. The yellow, green, and blue arrows indicate the increased centrilobular mitosis, hepatocellular necrosis and inflammatory cell infiltration, respectively. The yellow circle indicates hepatic cellular damage around the central vein. Scale bar, 100 μm. (TIFF 17697 kb)

Additional file 4: **Figure S4.** Confocal image of 3D HepaRG cells in the agarose hydrogel channel. (A) Agarose hydrogel gel containing HepaRG cells extracted from the polyolefin tube after 1 day of incubation. The cells in the gel were dyed with Calcein-AM (live, green) and EthD-1 (dead, red) and observed by confocal microscopy in the top, cross-section, and side views. Scale bar, 2 mm. (B) Cell viability of HepaRG cells on the monolayer (2D) and agarose hydrogel gel (3D) was measured by the CCK-8 assay after 1 day of incubation. (C) 3D HepaRG spheroids obtained by long-term cultivation (days 3, 6, and 11) in the agarose hydrogel gel from the polyolefin tube. Scale bar, 100 μm. (TIFF 7361 kb)

Additional file 5: **Figure S5.** Effects of H_2_O_2_ on zonal toxicity in HepaRG cells. Effects of H_2_O_2_ on cell viability, levels of CYP mRNAs, enzymatic activities of CYPs, and cytotoxicity of hepatotoxic drugs were evaluated using the same procedures performed in the CHIR-treated group. (A) Fully differentiated HepaRG cells were exposed to various concentrations of H_2_O_2_ for 10 days and the medium with the concentrated chemicals was replaced every 3 days. Cell viability was evaluated using CCK-8 assays after two washes with PBS. No change in the cross-reaction between H_2_O_2_ and the CCK-8 reagent was observed in the background values of controls. Levels of CYP mRNAs (*CYP2B6*, *CYP1A2*, *CYP2E1*, and *CYP3A4*) were analyzed in cells treated with H_2_O_2_ for 3 days using qRT-PCR. (B) The activities of CYP1A2 and CYP3A4 were measured using the P450-Glo CYP assay, and CYP2E1 activity was measured using HPLC-tandem mass spectrometry. *P < 0.05. (C) The hepatotoxic drugs tamoxifen, isoniazid, bromobenzene, and APAP were administered to HepaRG cells that had been pretreated with 200 μM H_2_O_2_ for 3 days. The viability of HepaRG cells was measured using the CCK-8 assay, and the dose-response curve was analyzed using GraphPad Prism software. (TIF 959 kb)

Additional file 6: **Table S1.** List of the primers used in this study. (DOCX 19 kb)

Additional file 7: **Table S2.** List of antibodies used in this study. (DOCX 13 kb)

## Abbreviation

3D: three dimensional
AhR: Aryl hydrocarbon receptor
AIF: apoptosis inducing factor
APAP: acetaminophen
CCK-8: Cell Counting Kit-9
CHIR: CHIR99021
CYP: cytochrome P450
DEGs: differentially expressed genes
DMSO: dimethyl sulfoxide
GSK3β: glycogen synthase kinase-3
PARP: poly (ADP-ribose) polymerase
PBS: phosphate buffered saline
PXR: Pregnane X receptors
TBS: Tris-buffered saline
