# Supplementary figures and images for: Human three-dimensional in vitro model of hepatic zonation to predict zonal hepatotoxicity
Source: J Biol Eng. 2019 Mar 6;13:22. doi: 10.1186/s13036-019-0148-5 (PMC6404355; doi:10.1186/s13036-019-0148-5)

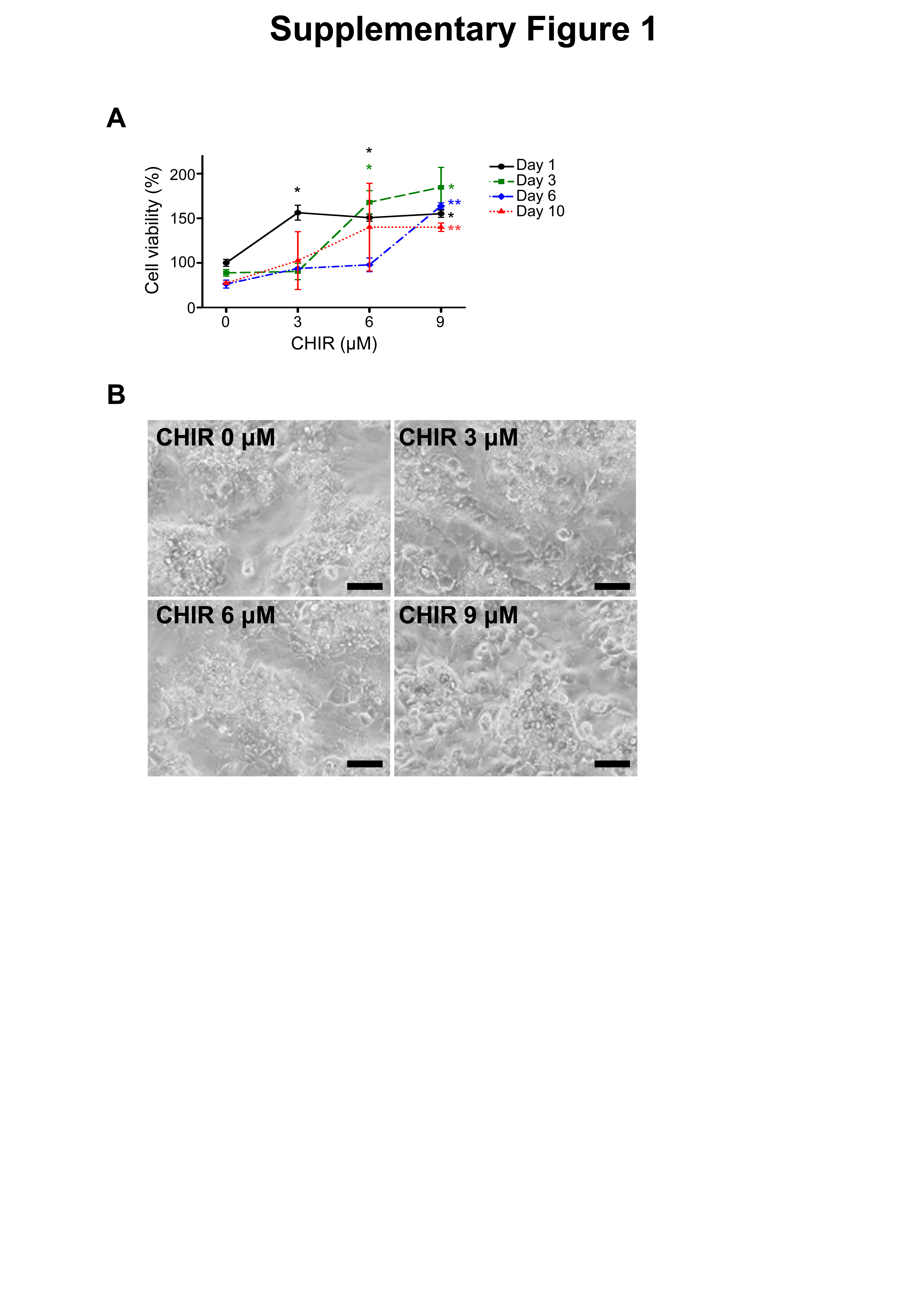

Supplement: Supplementary file 1 — Figure S1. Viability of monolayer-cultured HepaRG cells after CHIR treatment (days 1, 3, 6, and 10). (A) Cell viability was evaluated using CCK-8 assays on days 1, 3, 6, and 10 after the CHIR treatment. (B) HepaRG cells was observed before and after 3 days of CHIR treatment under a phase-contrast microscope (scale bar, 100 μm). The microscopic observation showed that fully differentiated HepaRG cells were organized in small clusters and displayed a typical hepatocytes-like morphology. (TIF 2598 kb) [file 13036_2019_148_MOESM1_ESM.tif]

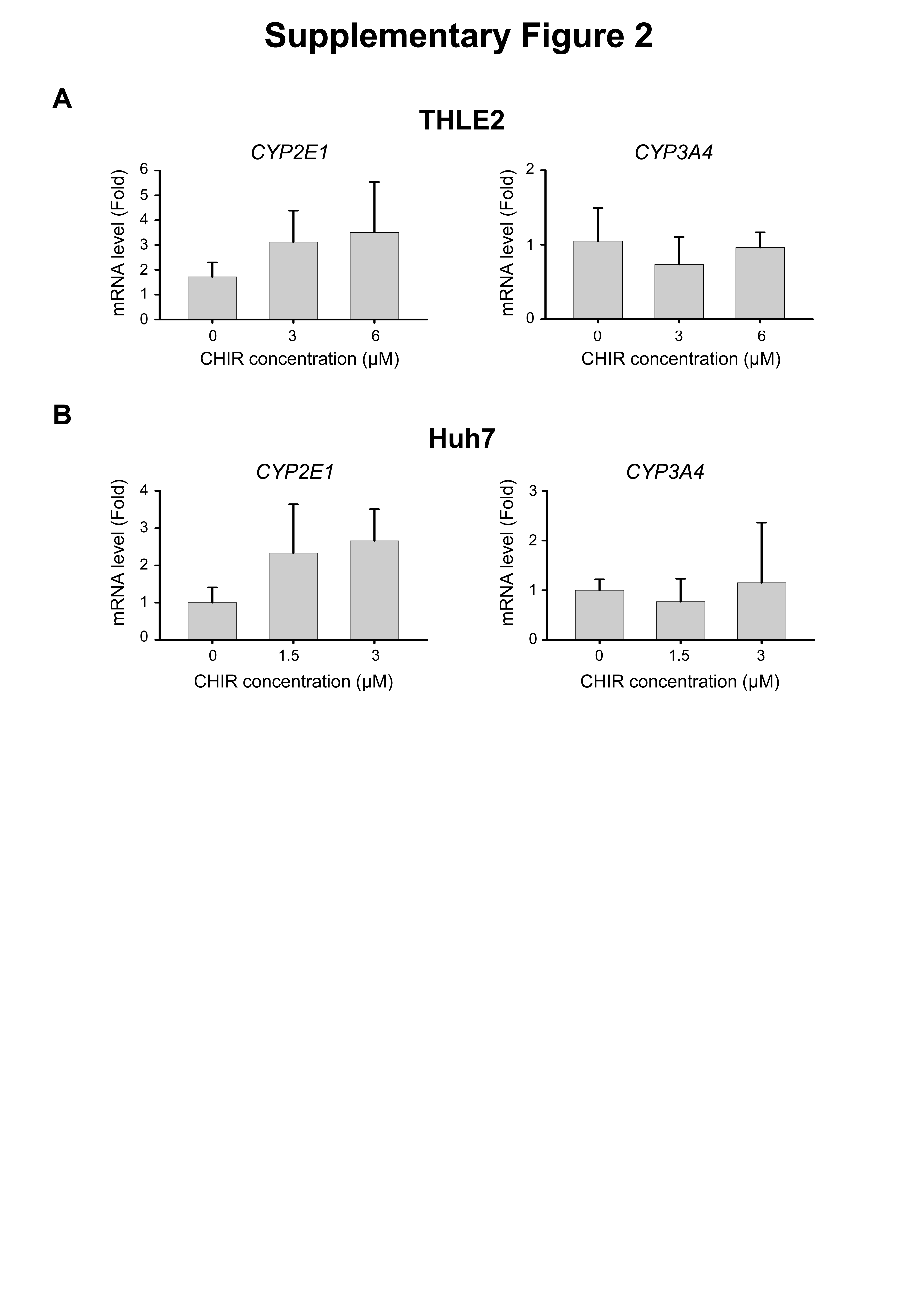

Supplement: Supplementary file 2 — Figure S2. qRT-PCR of CYP enzymes in various hepatocyte cell lines after CHIR treatment. The hepatocytes, including THLE2 (A) and Huh7 (B) were treated with CHIR for 3 days. The expression levels of zone-3-specific CYPs, such as CYP2E1 and CYP3A4, were evaluated by qRT-PCR. (TIF 532 kb) [file 13036_2019_148_MOESM2_ESM.tif]

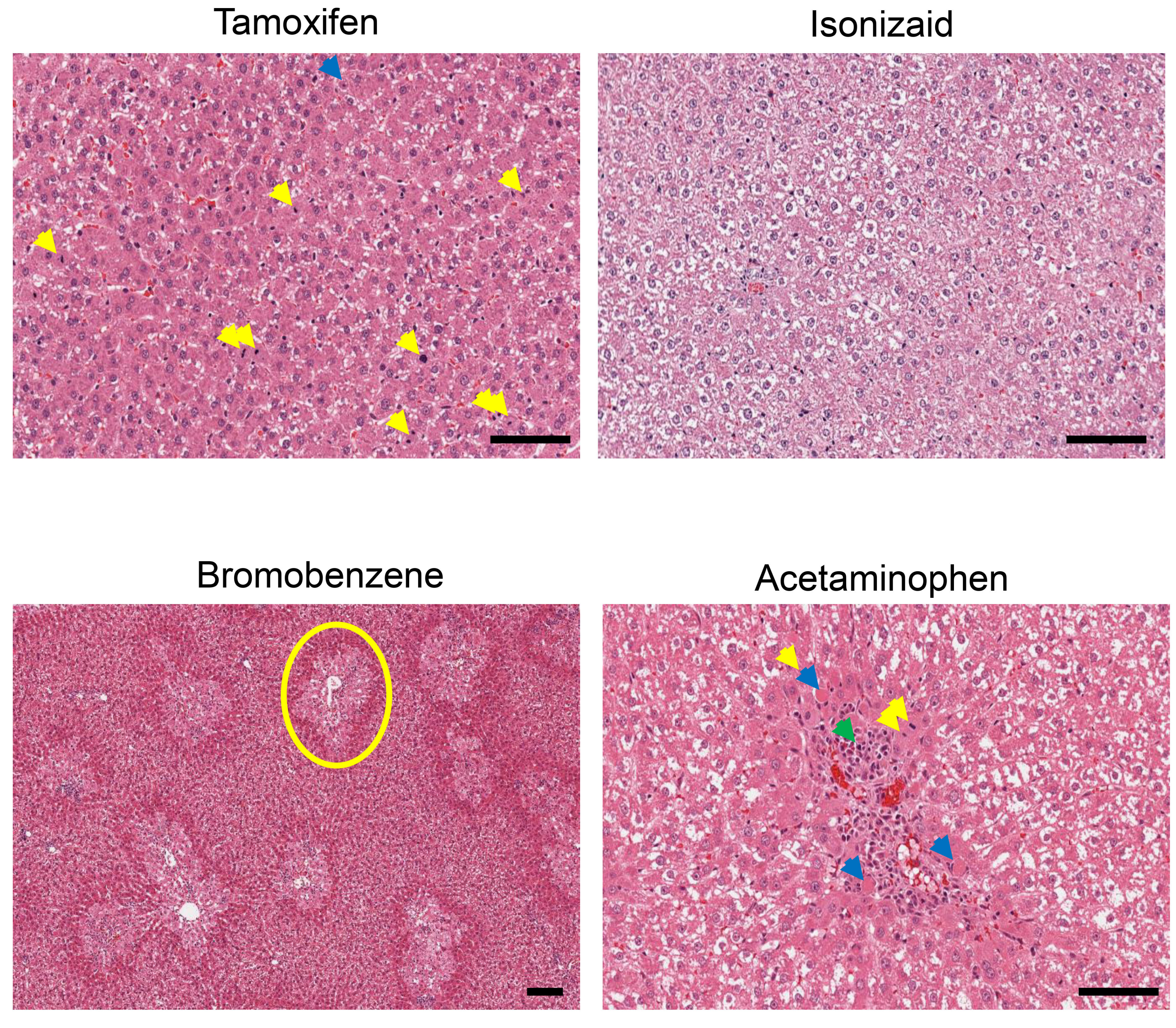

Supplement: Supplementary file 3 — Figure S3. Histopathologic observation of liver from rats treated with hepatotoxic drugs. The light microscopic image of the tamoxifen-, isoniazid-, bromobenzene-, and APAP-treated liver was obtained from the Open TG-GATEs database. The Sprague-Dawley rats were orally administered each drug for 29 days; tamoxifen, 20 mg/kg; isoniazid, 200 mg/kg; bromobenzene, 100 mg/kg; and APAP, 1000 mg/kg. The yellow, green, and blue arrows indicate the increased centrilobular mitosis, hepatocellular necrosis and inflammatory cell infiltration, respectively. The yellow circle indicates hepatic cellular damage around the central vein. Scale bar, 100 μm. (TIFF 17697 kb) [file 13036_2019_148_MOESM3_ESM.tiff]

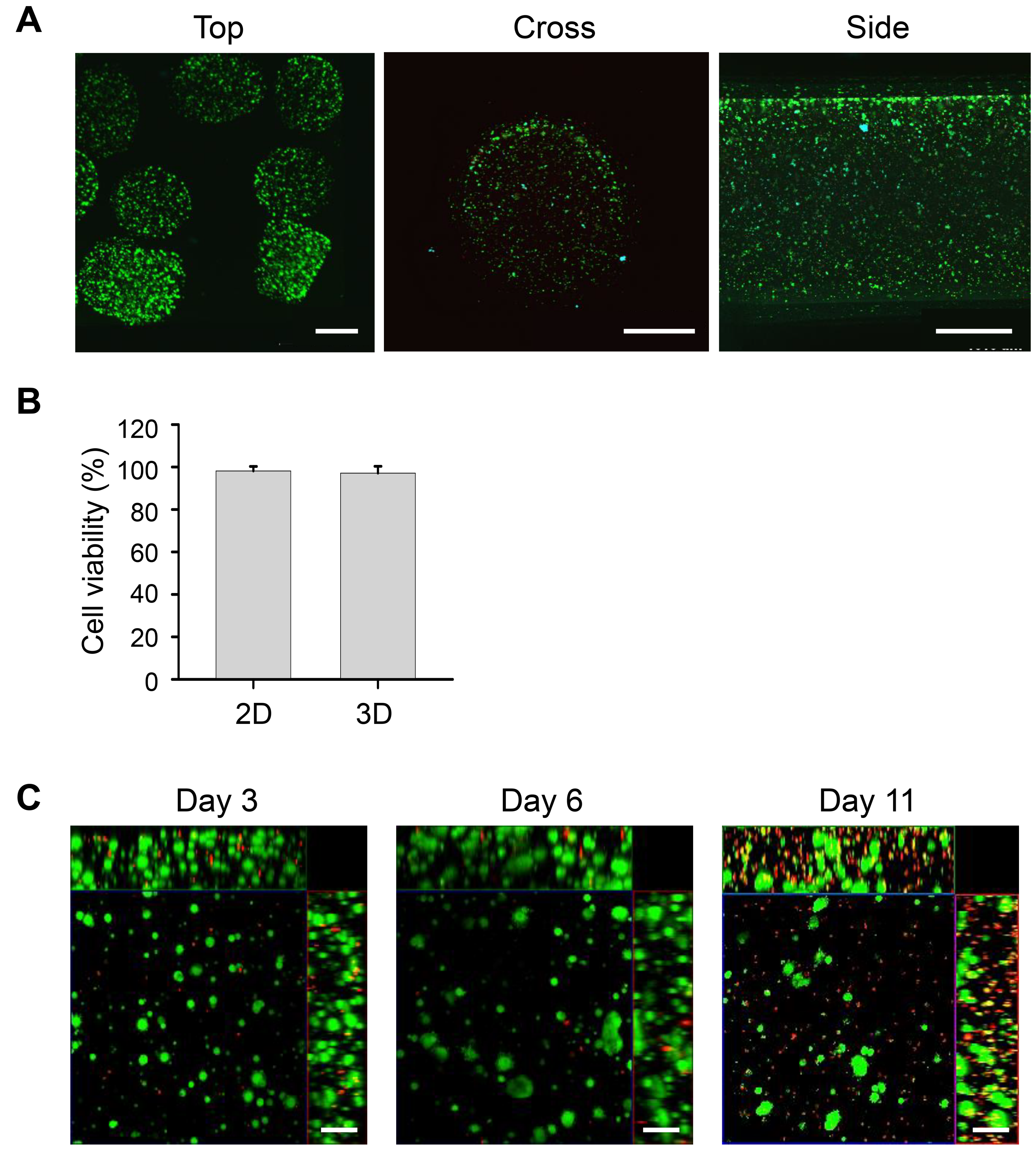

Supplement: Supplementary file 4 — Figure S4. Confocal image of 3D HepaRG cells in the agarose hydrogel channel. (A) Agarose hydrogel gel containing HepaRG cells extracted from the polyolefin tube after 1 day of incubation. The cells in the gel were dyed with Calcein-AM (live, green) and EthD-1 (dead, red) and observed by confocal microscopy in the top, cross-section, and side views. Scale bar, 2 mm. (B) Cell viability of HepaRG cells on the monolayer (2D) and agarose hydrogel gel (3D) was measured by the CCK-8 assay after 1 day of incubation. (C) 3D HepaRG spheroids obtained by long-term cultivation (days 3, 6, and 11) in the agarose hydrogel gel from the polyolefin tube. Scale bar, 100 μm. (TIFF 7361 kb) [file 13036_2019_148_MOESM4_ESM.tiff]

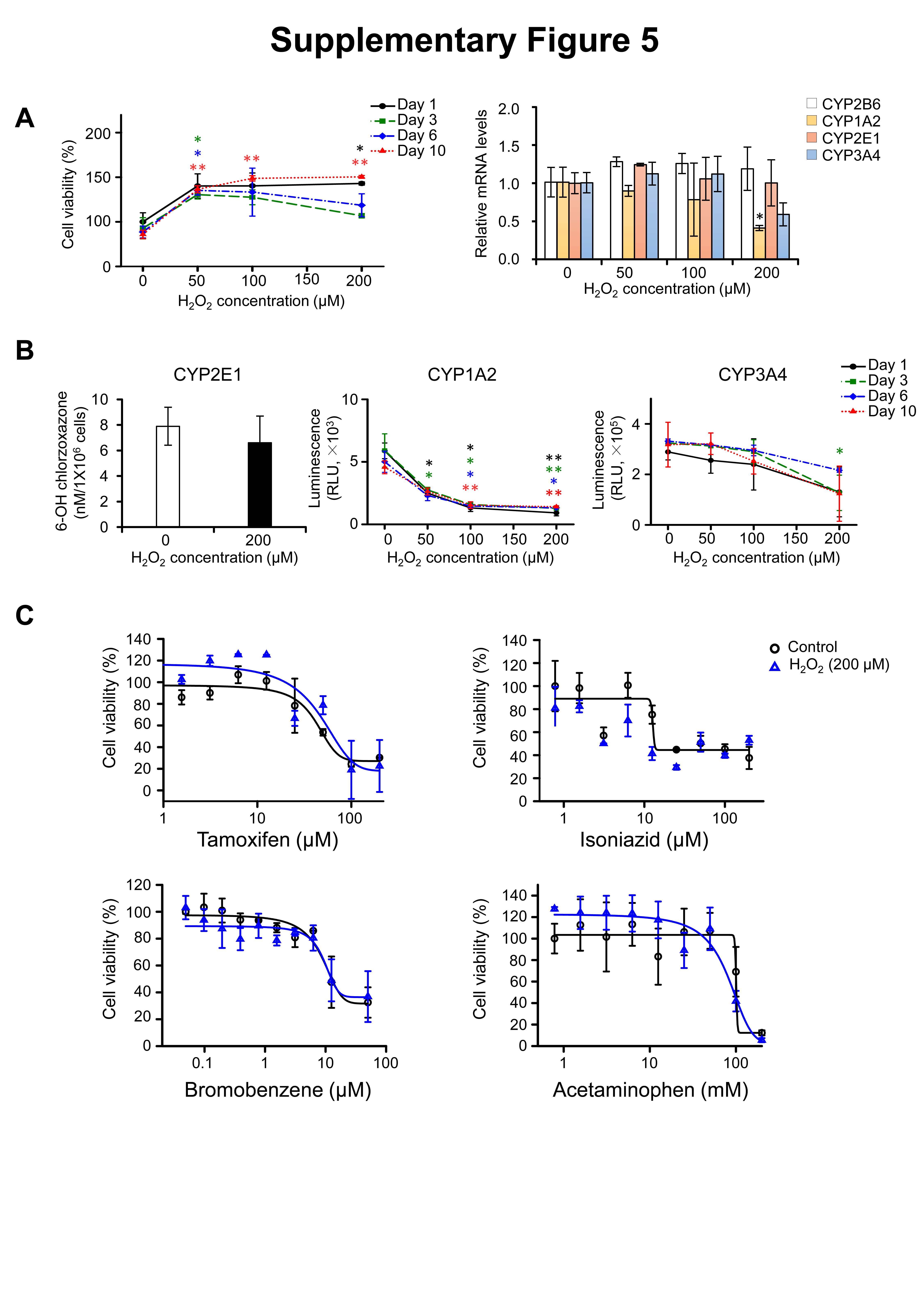

Supplement: Supplementary file 5 — Figure S5. Effects of H2O2 on zonal toxicity in HepaRG cells. Effects of H2O2 on cell viability, levels of CYP mRNAs, enzymatic activities of CYPs, and cytotoxicity of hepatotoxic drugs were evaluated using the same procedures performed in the CHIR-treated group. (A) Fully differentiated HepaRG cells were exposed to various concentrations of H2O2 for 10 days and the medium with the concentrated chemicals was replaced every 3 days. Cell viability was evaluated using CCK-8 assays after two washes with PBS. No change in the cross-reaction between H2O2 and the CCK-8 reagent was observed in the background values of controls. Levels of CYP mRNAs (CYP2B6, CYP1A2, CYP2E1, and CYP3A4) were analyzed in cells treated with H2O2 for 3 days using qRT-PCR. (B) The activities of CYP1A2 and CYP3A4 were measured using the P450-Glo CYP assay, and CYP2E1 activity was measured using HPLC-tandem mass spectrometry. *P < 0.05. (C) The hepatotoxic drugs tamoxifen, isoniazid, bromobenzene, and APAP were administered to HepaRG cells that had been pretreated with 200 μM H2O2 for 3 days. The viability of HepaRG cells was measured using the CCK-8 assay, and the dose-response curve was analyzed using GraphPad Prism software. (TIF 959 kb) [file 13036_2019_148_MOESM5_ESM.tif]
