# Supplementary material for: Human three-dimensional in vitro model of hepatic zonation to predict zonal hepatotoxicity
Source: J Biol Eng. 2019 Mar 6;13:22. doi: 10.1186/s13036-019-0148-5 (PMC6404355; doi:10.1186/s13036-019-0148-5)
Supplement: Supplementary file 6 — Table S1. List of the primers used in this study. (DOCX 19 kb) [file 13036_2019_148_MOESM6_ESM.docx]

**Supplementary Table 1.** List of the primers used in this study.

| Gene | Primer (Forward) | Primer (Reverse) | Reference |
| --- | --- | --- | --- |
| *CYP1A2* | TCCTTCGCTACCTGCCTAAC | AGGCATTCAGGGAAGGGTTG | NM_000761 |
| *CYP2B6* | TTGCTACTCCTGGTTCAGCG | GTCCCAGGTGTACCGTGAAG | NM_000767 |
| *CYP2E1* | TTGAAGCCTCTCGTTGACCC | CGTGGTGGGATACAGCCAA | NM_000773 |
| *CYP3A4* | CCAAGCTATGCTCTTCACCG | TCAGGCTCCACTTACGGTGC | NM_017460 |
| *AXIN2* | CCTAAAGGTCGTGTGTGGCT | ACAGTTTCCGTGGACCTCAC | XM_025362285 |
| *ALB* | TTTGGTTAGGCTAGGGCTTA | CATCACTTACTTGGATGGCT | M12523 |
| *18S rRNA* | GTAACCCGTTGAACCCCATT | CCATCCAATCGGTAGTAGCG | NR_145820 |
