# Supplementary material for: Human three-dimensional in vitro model of hepatic zonation to predict zonal hepatotoxicity
Source: J Biol Eng. 2019 Mar 6;13:22. doi: 10.1186/s13036-019-0148-5 (PMC6404355; doi:10.1186/s13036-019-0148-5)
Supplement: Supplementary file 7 — Table S2. List of antibodies used in this study. (DOCX 13 kb) [file 13036_2019_148_MOESM7_ESM.docx]

**Additional file 7: Table S2.** List of antibodies used in this study

| Antibodies | Catalog No. | Company | Dilution |
| --- | --- | --- | --- |
| β-catenin | 4270 | Cell Signaling | 1:1000 for WB |
| CYP2E1 | Ab28146 | Abcam | 1:1000 for WB |
| AIF | 4642 | Cell Signaling | 1:1000 for WB |
| PARP | 9542 | Cell Signaling | 1:1000 for WB |

*WB: Western blotting
